# Psychometric properties of the effort-reward imbalance scale in Chinese version for university students

**DOI:** 10.3389/fpsyg.2023.1128290

**Published:** 2023-02-01

**Authors:** Chu Kequn, Li Biao, She Shaohua

**Affiliations:** College of Educational Science, Guangxi Science and Technology Normal University, Guangxi, China

**Keywords:** effort-reward imbalance, overcommitment, university students, validity, reliability

## Abstract

**Objective:**

The main purpose of the study was to translate the Effort-Reward Imbalance Scale for University Students (ERIUS) and assess its psychometric properties in the Chinese cultural context.

**Methods:**

We translated the original English version of the ERIUS into Chinese and undertook exploratory and confirmatory factor analysis using data collected from university students. The Stress Scale for College Students was selected as the criterion measure to examine the association between psychological stress and effort-reward imbalance. The validity and reliability of the translated version of the ERIUS were also assessed, and a sub-sample of participants (*n* = 120) completed the measure twice, with a two-week interval to assess test–retest reliability.

**Result:**

Results of the exploratory factor analysis using data from 314 students showed that the Chinese version of the ERIUS had 14 items and 3 factors: effort, reward and overcommitment. Confirmatory factor analysis using data from 584 students showed that the Chinese version of the ERIUS had adequate structural validity (*χ*^2^ = 107.10, df = 32, RMSEA = 0.08, NFI = 0.90, CFI = 0.91, GFI = 0.90, PGFI = 0.62).

**Conclusion:**

The Chinese version of the Effort-Reward Imbalance Scale for University Students has adequate psychometric properties in the Chinese cultural context and can be used as an effective tool to measure psychosocial stress of university students in China.

## Introduction

Many research studies show that the psychosocial pressure environment seriously affects the mental health, academic performance, and future career development of university students (Adams, 2004; Bernhardt et al., 2012; Singh et al., 2012; Fanning, 2016). Psychosocial pressure environments refer to social environments that cause individuals to experience psychological pressure, mainly regarding the social environment as the source of psychological pressure (Jiang and Peng, 2006). Existing research shows that current university students need to spend a lot of time, energy, and emotion due to increasingly challenging courses and demanding tasks. They also experience a lack of respect from society and face a serious employment situation, which creates a poor psychosocial environment and increases the psychosocial pressures facing them (Akinola and Oladunmoye, 2018; Collin et al., 2020). Research also shows that a negative psychosocial stress environment has a significant predictive effect on negative emotions, fatigue, physical pain, low self-evaluation, and suicidal tendencies of university students (Låftman et al., 2015).

At present, the diagnosis of psychosocial stress depends on behavioral evaluation, due to the lack of biomarkers to objectively identify psychosocial stress among university students. However, there are few measurement tools available to explore the source of university students’ group psychological pressure, which limits the development of research to a certain extent. Therefore, in a complex psychological and social environment, it is important to identify the components that cause university students’ psychological and social pressure. In the study of occupational health, several theoretical models of stressful psychosocial work environments have been developed and applied. These include the work-family balance model, work environment matching model, effort-reward imbalance model and work demand-control model (Siegrist, 1996; Siegrist et al., 2004; Elovainio et al., 2010; Chungkham et al., 2013). One of the most widely tested models, the ‘effort-reward imbalance model’ (ERI), posits that the imbalance between high effort and low reward is the cause of work stress, which will be more obvious when individuals are overcommitted (Siegrist, 1996; Siegrist et al., 2004). Many studies have shown that an effort-reward imbalance leads to various physical discomforts and psychological diseases (Hinz et al., 2016; Siegrist, 2016). At present, researchers have retained the basic principle of the effort-reward imbalance model and migrated the model to university settings. And revised the effort-reward imbalance scale for university students, namely the effort-reward Imbalance for University Students (ERIUS; Wege et al., 2017). The scale has been revised and verified in other countries, with high reliability and validity, and is a useful measure of the source of psychological and social stress in university students (Williams et al., 2018; Portoghese et al., 2019; Porru et al., 2021). Studies have also shown that the effort-reward imbalance of university students can significantly predict their burnout, fatigue, low self-evaluation, and suicidal tendencies (Wahrendorf et al., 2012; Shang et al., 2014; Wege et al., 2017; Hwang et al., 2019).

Since there is no Chinese version of the scale, this study attempted to revise the ERIUS in Chinese and assess its reliability and validity. The scale was revised in a sample of university students under the Chinese cultural context, and we used exploratory factor analysis and confirmatory factor analysis to evaluate its psychometric characteristics, to provide a reliable measurement tool for Chinese related research and promote the development of research in this field in China. Some studies also show that there is a significant positive correlation between effort-reward imbalance and stress performance among university students. The degree of effort-reward imbalance in university students has been shown to positively predict their stress performance (Williams et al., 2018; Portoghese et al., 2019; Porru et al., 2021). Therefore, this study took stress as the criterion validity to explore the relationship between effort-reward imbalance and stress in university students.

## Method

### Translation

Before revision, we contacted the original author of the Effort-Reward Imbalance for University Students scale, obtained authorization, and translated it using the back-translation method. The original English version of ERIUS was translated into Chinese according to standard guidelines, which are widely accepted to successfully translate measures in cross-cultural research (Behr, 2017). First, two translators were asked to independently translate the English version of the scale into Chinese to develop a preliminary Chinese version of the scale. The research group subsequently held two rounds of meetings to discuss the translation until consensus was reached. Then, two native English speakers (bilingual in English and Chinese) were invited to translate Chinese into English. Before all translators and researchers came to an agreement, any differences between the original version and the back translated version had been discussed. Then, using the original version, the preliminary Chinese version and the back translated English version of the scale, the Chinese version of the Effort Reward Imbalance for University Students was formed by comparing the items one by one and considering the words used. The final items and scoring method of the scale were considered consistent with the original questionnaire.

### Participants

We used cluster sampling method to sample university students from four universities in Guangxi, China in the study. The data were collected through an online cross-sectional survey in October 2022. The main examiner in charge of student affairs first contacted the students from the four universities, and then all students received a short invitation and a link to an online questionnaire through WeChat (a multi-functional SMS mobile application). The questionnaire consisted of two parts, the first part introduced the investigation and consent form. The second part was the Chinese version of The Effort-Reward Imbalance for University Students. The study did not commence until the student fully completed the consent form and agreed to participate. The sample size met the requirements of factor analysis and other psychometric assessments (Floyd and Widaman, 1995), and the sample was divided into prediction samples, formal samples, and test–retest samples.

Sample 1 (prediction sample, used for exploratory factor analysis): a simple and convenient sampling method was adopted, and the questionnaire was distributed in the form of online. A total of 330 university students were selected from four universities in Guangxi Province, and 314 valid questionnaires were recovered, yielding an effective response rate of 95.3%. Of them, there were 192 males and 122 females with an age range of 18–24 years.

Sample 2 (the formal samples, used for confirmatory factor analysis and reliability assessment): The questionnaire was distributed online. A total of 610 university students were selected from six universities in Guangxi, and 584 valid questionnaires were received, providing an effective response rate of 95.7%. Of them, there were 310 males and 274 females with an age range of 17 ~ 25 years. Two weeks later, 120 of Sample 2 were randomly selected as the test–retest sample that included 63 males and 57 females.

### Measures

#### The effort-reward imbalance

We used the Chinese version of the ERIUS to measure the effort-reward ratio and overcommitment. The ERIUS includes three factors and 14 items in total, specifically: the effort factor is measured by 3 items, reward is measured by 6 items, and overcommitment is measured by 5 items. Items in the scale are answered using a 4-point Likert scale (1‘strongly disagree’, 2‘disagree’, 3‘agree’, 4‘strongly agree’). Effort-reward ratio was computed by dividing the ‘effort’ score by the ‘reward’ score, using the established algorithm.

#### Stress

We used the Chinese version of the Stress Scale for university Students (SSCS) as a tool to measure stress. Which has adequate statistical indicators among Chinese college students (Li and Mei, 2002). The scale includes three factors, which mainly measure the performance of university students’ psychological stress, such as learning annoyance, personal annoyance, and negative life. The scale consists of 34 items rated using a 5-point Likert scale (1 = strongly disagree, 5 = strongly agree). In this study, Cronbach α of the scale was 0.89.

#### Data analysis

SPSS 22.0 was used for exploratory factor analysis and to assess the scale’s internal consistency (Cronbach *α* Coefficient), retest reliability, split-half reliability, and criterion related validity. Amos 22.0 was used for confirmatory factor analysis.

## Results

We used Harman’s single-factor test to control common method bias. This resulted in nine components explaining 83.75% of the variance, with the first one explaining only 30.65%. Therefore, there is no common method bias in this study.

### Exploratory factor analysis

First, exploratory factor analysis was conducted using sample 1’s responses. The Kaiser–Meyer–Olkin (KMO) value was 0.83 > 0.5 using principal component analysis and Promax oblique rotation methods, and results of the Bartlett’s sphericity test showed: *x*^2^ = 2,504.59, *p* < 0.01. Hence, further statistical analysis could be conducted. In combination with the gravel map, three main factors (Table 1) were obtained, including effort, reward and overcommitment.

**Table 1:** Factor load of each item—Results of exploratory factor analysis.

Effort (Eff1–Eff3)		Reward (Rew1–Rew6)		Overcommitment (Oc1–Oc5)	
Item	Factor loading	item	Factor loading	item	Factor loading
eff1	0.89	Rew1	0.88	Oc1	0.92
Eff2	0.86	Rew2	0.89	Oc2	0.83
Eff3	0.91	Rew3	0.92	Oc3	0.82
		Rew4	0.86	Oc4	0.87
		Rew5	0.91	Oc5	0.81
		Rew6	0.90		

### Confirmatory factor analysis

**Figure 1:**
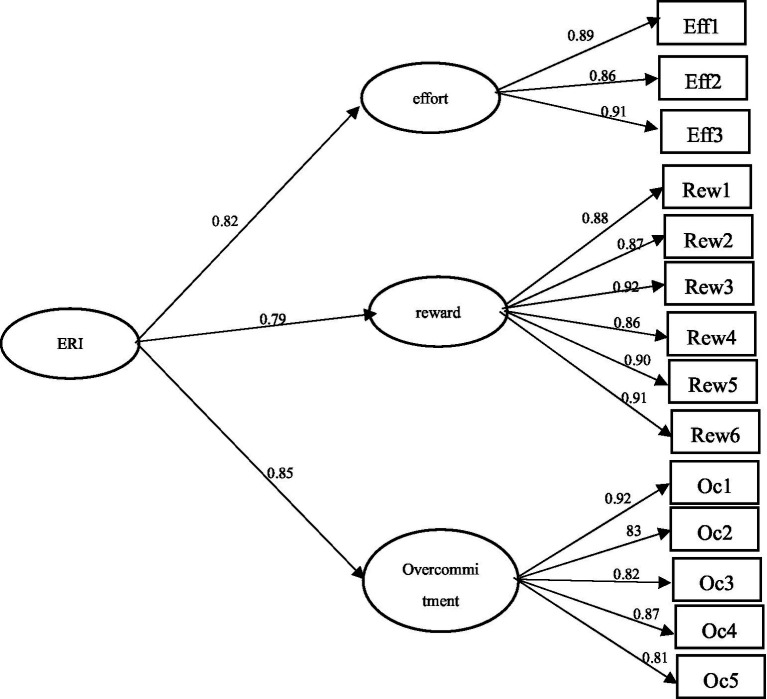
Confirmatory factor analysis of model fit.

We used Amos 22.0 to perform confirmatory factor analysis using sample 2’s responses. The results showed that each fitting index was consistent with the statistical indicators: *χ*^2^ = 107.10, df = 32, RMSEA = 0.08, NFI = 0.90, CFI = 0.91, GFI = 0.90, PGFI = 0.62, indicating that the model had high structural validity (see Figure 1).

### Reliability assessment

A Reliability assessments were performed using sample 2’s responses.

### Internal consistency reliability

Cronbach *α* is usually used to assess internal consistency reliability. Generally, a Cronbach’s alpha of greater than 0.7 indicates that the internal consistency reliability of the scale is adequate (Floyd and Widaman, 1995). Results showed that Cronbach’s alpha for the three ERIUS subscales were 0.87, 0.89, and 0.91, respectively, indicating adequate internal consistency reliability was adequate.

### Split half reliability

The items of the three subscales were divided into odd and even halves, and split half reliability was calculated according to the Spearman Brown formula. The results showed that the split half reliability of the three subscales of effort, reward and overcommitment was 0.88, 0.91and 0.85 respectively, which indicates adequate reliability.

### Test–retest reliability

Two weeks later, 120 participants were randomly selected from samples 2 as retest samples, and the correlation coefficient with samples 2 ranged between 0.84 and 0.90, with the test–retest reliability for the total scale being 0.86. In addition, the test–retest reliability for the three subscales was 0.84, 0.90, and 0.85, respectively.

### Criterion validity assessment

We used Pearson correlation analysis to investigate the relationships between the ERIUS and the SSCS. The results showed that the Effort-reward ratio and stress scores were significantly positively correlated (*r* = 0.57, *p* < 0.001), and the correlation coefficient between overcommitment scores and stress scores was 0.46 (*p* < 0.01), indicating adequate criterion validity (Appendix).

## Discussion

The main purpose of this study was to assess the psychometric properties of the Chinese version of the ERIUS in the Chinese cultural context. This study revised the Chinese version of ERIUS through translation, exploratory factor analysis, reliability and validity testing and confirmatory factor analysis. Results of exploratory factor analysis and item analysis indicated that the commonality of the 14 items was statistically significant. Therefore, all items and factor structure included in the revised Chinese version of the scale are consistent with the original scale. The internal consistency coefficients of the overall scale and the three factors were all above 0.87, indicating adequate reliability.

Factor analysis is a recognized method to assess the potential structure of a questionnaire, and was used to investigate the factor structure of the Chinese version of the ERIUS in this study. Exploratory factor analysis was conducted and the shapes of the gravel map and the factor load map showed that all three factors of the original scale could be retained in the revised version. The 14 items of the ERIUS have statistically significant commonality, so the scale’s original 14 items can also be retained. The results of confirmatory factor analysis showed that ERIUS’ three factors had a good fit, and all statistical indicators were within an acceptable range. The adaptability, reliability and validity of the model were also within an acceptable range. In addition, the internal consistency coefficient of the three subscales were 0.87, 0.89, and 0.91, respectively, indicating adequate reliability. Our results also showed that the effort-reward ratio of the ERIUS and the Stress Scale for university students were significantly positively correlated (Williams et al., 2018; Portoghese et al., 2019; Porru et al., 2021). Some previous results have shown that the higher the ratio of effort-reward imbalance, the greater the psychological pressure of university students (Porru et al., 2021). We also found that the overcommitment subscale of ERIUS and the stress scale were significantly positively correlated, which was consistent with previous findings (Portoghese et al., 2019). Overall, our findings show that the Chinese version of the Effort-Reward Imbalance for University Students Scale has high criterion validity and the scale has adequate stability over time.

One limitation should be mentioned. The results are limited to a sample of university students from Guangxi, China, and it is not known whether they can be generalized to students in other provinces. Moreover, we were not in a position to analyze potential selection bias within the confines of this study.

## Conclusion

we were able to demonstrate satisfactory psychometric properties of a short, theory based self-report assessment of stressful working environments of university students. To sum up, this study demonstrated that the Chinese version of the Effort-Reward Imbalance for University Students Scale has adequate psychometric properties, which can be used as a tool to evaluate university students’ effort-reward imbalance.

## Data availability statement

The raw data supporting the conclusions of this article will be made available by the authors, without undue reservation.

## Author contributions

CK is responsible for project implementation, experiment design, and thesis writing. LB is responsible for data analysis and statistical results. SS is responsible for project supervision and thesis revision. All authors contributed to the article and approved the submitted version.

## Funding

This research was supported by the Guangxi Science and Technology Normal University Youth Scientific Research Innovation Team Project [Title: Teachers’ Professional Mental Health Research Team (GXKS2020QNTD05)].

## Conflict of interest

The authors declare that the research was conducted in the absence of any commercial or financial relationships that could be construed as a potential conflict of interest.

## Publisher’s note

All claims expressed in this article are solely those of the authors and do not necessarily represent those of their affiliated organizations, or those of the publisher, the editors and the reviewers. Any product that may be evaluated in this article, or claim that may be made by its manufacturer, is not guaranteed or endorsed by the publisher.

## Appendix

The Chinese version and the original English version of the effort-reward imbalance scale for University students.

**Table:**

付出 (Effort)	回报 (reward)	过度投入 (overcommitment)	
1繁重的学习任务时常让我感到时间压力。 I have constant time pressure due to a heavy study load	4我会从监督者(老师)那里受到应有的尊重。 I receive the respect I deserve from my supervisors (teachers)	10早上一起床，我就开始考虑学习的事。 As soon as I get up in the morning I start thinking about study problems	
2当我正在准备考试的时候，时常会被外界干扰。 I have many interruptions and disturbances while preparing for my exams	5我会从同学那里受到应有的尊重。 I receive the respect I deserve from my fellow students	11当我回到家我可以很容易的放松下来。 When I get home, I can easily relax and “switch off” from study	
3我的学习负担越来越大。 My study load has become more and more demanding	6我大学里，我没有遭受不剬平对待。 I am treated unfairly at university	12身边的朋友都说我对学业牺牲了太多。 People close to me say I sacrifice too much for my study	
	7我不确定是否顺利完成大学阶段的学习。 I am not sure whether I can successfully accomplish my university trainings	13 当我上床睡觉的时候，仍然想着学生工作。 Student work rarely lets me go; it is still on my mind when I go to bed	
	8 就我的努力而言，我得到了应有的回报。 Considering all my efforts, I receive the appreciation that I deserve	14 如果我推迟一些本来应该今天做完的事，我将难以入睡。 If I postpone something that I was supposed to be done today I’ll have trouble sleeping at night	0.81
	9 考虑到我的努力和成绩，我将会有很好的就业前景。 Considering all my efforts and achievements, my job promotion prospects are adequate		
